# Dual functions of the *Aedes aegypti* ecdysone receptor in dengue virus replication and reproduction control

**DOI:** 10.1186/s13071-026-07298-0

**Published:** 2026-03-22

**Authors:** Shih-Che Weng, Yu-Shu Lin, Po-Nien Tsao, Shin-Hong Shiao

**Affiliations:** 1 Department of Tropical Medicine and Parasitology, College of Medicine, National Taiwan University, Taipei, Taiwan; 2 Department of Pediatrics, National Taiwan University Hospital, Taipei, Taiwan; 3 Research Center for Developmental Biology and Regenerative Medicine National Taiwan University, Taipei, Taiwan

**Keywords:** *Aedesaegypti*, Dengue virus, Ecdysone receptor, Vitellogenesis, Antiviral

## Abstract

**Background:**

The ecdysone receptor (*EcR*) is a central regulator of mosquito physiology, best known for its role in vitellogenesis. However, its contribution to antiviral defense and dengue virus (DENV) replication in *Aedes aegypti* remains poorly understood.

**Methods:**

RNA interference was used to silence *Aedes aegypti EcR* (*AaEcR*). Effects on DENV replication, immune gene expression, ovarian development, vitellogenin (Vg) synthesis, and target of rapamycin (TOR) pathway activity were assessed using molecular, cellular, and phenotypic analyses.

**Results:**

Silencing *AaEcR* markedly suppressed DENV replication, viral protein expression, and virion production. These antiviral effects coincided with increased expression of antimicrobial peptides and activation of innate immune pathways, indicating that *AaEcR* facilitates viral replication by dampening host defenses. In addition, *AaEcR* proved essential for reproductive output. Knockdown impaired ovarian development, reduced follicle size and number, and lowered egg production by ~30%, although egg viability was unaffected. At the molecular level, *AaEcR* depletion strongly reduced *Vg* transcription and protein abundance, along with decreased phosphorylation of S6 kinase, suggesting that *AaEcR* promotes fecundity through both transcriptional activation and TOR-Vg signaling.

**Conclusions:**

*AaEcR* functions as a dual regulator of mosquito biology, suppressing antiviral immunity while enhancing reproductive output. This tradeoff between immunity and fecundity highlights *AaEcR* as a promising molecular target for vector control. Disrupting *EcR* signaling could simultaneously reduce mosquito population size and limit arboviral transmission, offering a potential strategy for integrated management of mosquito-borne diseases.

**Graphical Abstract:**

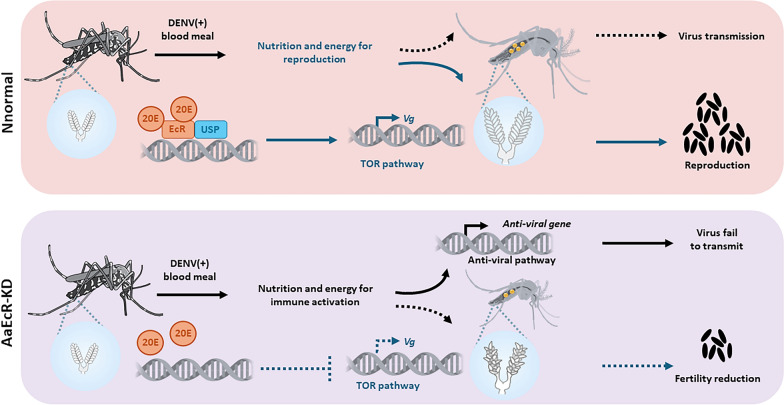

**Supplementary Information:**

The online version contains supplementary material available at 10.1186/s13071-026-07298-0.

## Background

Global initiatives to control vector-borne diseases, primarily those transmitted by mosquitoes, aim to reduce disease incidence through strategies such as insecticide application and the management of mosquito breeding habitats [1]. However, despite decades of implementation, these approaches have not been effective in curtailing disease transmission or preventing outbreaks. Furthermore, current strategies face growing challenges, including the emergence of insecticide-resistant malaria and *Aedes* mosquito strains [2–5]. The absence of effective treatments or reliable vaccines for dengue fever and Zika syndrome highlights the need for alternative control methods [6–10].

The dengue virus, primarily transmitted by *Aedes* mosquitoes, is a single-stranded positive-sense RNA virus belonging to the *Flavivirus* genus within the Flaviviridae family [10, 11]. It is classified into four serotypes: DENV1, DENV2, DENV3, and DENV4. The approximately 11 kilobase genomic RNA of the dengue virus contains a single open reading frame encoding three structural proteins (capsid (C), envelope (E), and precursor membrane (prM)) and at least seven nonstructural (NS) proteins (NS1, NS2A, NS2B, NS3, NS4A, NS4B, and NS5). The structural proteins assemble with the viral genome to form virions, while the nonstructural proteins facilitate viral RNA replication [10, 11].

Immune signaling pathways protect mosquitoes from infections caused by pathogens and microorganisms [12–14]. The three major immune signaling pathways are the Toll, immune deficiency (IMD), and Janus kinase (JAK)/signal transducer and activator of transcription (STAT) pathways, which trigger immune effector molecules such as antimicrobial peptides (AMPs). Additionally, the RNA interference (RNAi) pathway is critical in defending against viral attacks [15]. The Toll pathway in mosquitoes regulates AMP expression via the transcription factor Rel1 and is induced by fungi, Gram-positive bacteria, and viruses. The JAK-STAT pathway exhibits antiviral activity against DENV in *Aedes aegypti* and is activated by ligand binding to the Domeless (*Dome*) receptor [16, 17]. The IMD pathway primarily controls AMP gene induction by Gram-negative bacteria but also plays a minor role in fighting viral infections in *Aedes* mosquitoes [18, 19]. RNAi is the main antiviral pathway, and impairment of this pathway leads to increased viral titer [20]. Together, these pathways constitute the core of the mosquito’s innate immune system.

The triggering of vitellogenesis in anautogenous female mosquitoes by the consumption of a blood meal is pivotal in the reproductive process. An enteric signal from the midgut stimulates the brain to release ovarian ecdysteroidogenic hormone (*OEH*), which then binds to its cognate receptor (*OEHR*) on ovarian follicle cells. This interaction, in conjunction with amino acid signaling, activates the target of rapamycin- (*TOR*) and insulin-signaling pathways, leading to the conversion of cholesterol into ecdysteroids. The secreted ecdysone is subsequently converted into the active hormone 20-hydroxyecdysone (20E) by cytochrome P450 314A1 (*CYP314A1*) in the fat body. Circulating 20E binds to a heterodimeric receptor complex composed of the ecdysone receptor (*EcR*) and ultraspiracle protein (*USP*), initiating the activation of early response genes that regulate the synthesis of yolk protein precursors (YPPs) in the fat body, including vitellogenin (Vg), vitellogenin carboxypeptidase (VCP), vitellogenic cathepsin B (VCB), and lipophorin (Vp). These YPPs are subsequently incorporated into developing oocytes, a process essential for ovarian maturation and offspring viability [21–24].

The 20E signaling pathway plays a critical role throughout the mosquito life cycle, governing metabolic processes, development, and reproduction. The digestion of a blood meal triggers metabolic processes that are largely regulated by 20E signaling. Silencing *AaEcR* expression results in the reduced expression of glycolysis and glycogen-metabolism-related genes, and in the fat body, influences lipid metabolism. *AaEcR* expression in the fat body peaks during vitellogenesis (18–24 h post-blood meal, hPBM). Silencing *AaEcR* in female mosquitoes leads to smaller ovarian follicles, reduced egg production, and developmental abnormalities [25–27].

Although previous studies have reported on the involvement of *EcR* in mosquito development and reproduction, a more comprehensive understanding of how 20E signaling influences the allocation of resources between oviposition and pathogen defense is needed. This study aims to clarify the regulatory role of *AaEcR* in reproductive processes and dengue virus replication. The central hypothesis posits that *AaEcR* influences both reproduction and viral replication through the modulation of immune responses, making it a prospective target for strategies to control mosquito propagation and the transmission of flaviviruses.

## Methods

### Mosquito rearing and feeding

*Aedes aegypti* (UGAL/Rockefeller strain) mosquitoes were reared at 28 ℃ and under a 12-h light/dark cycle. Eggs collected on filter paper were incubated in aerated water for 30–60 min within a vacuum environment. Hatched larvae were then evenly distributed into plastic trays filled with dechlorinated water. Growing larvae were fed yeast extract daily on the basis of their developmental stage, and turbid water was replaced with fresh as required. Pupae were separated from fourth instar larvae and housed in insect rearing cages. Emerged adults were maintained on 10% sterile sucrose solution, which was absorbed by a cotton ball. Female mosquitoes aged 3–5 days were used for the following experiment. To maintain the required number of mosquitoes for our study, 5- to 7-day-old female mosquitoes were starved for 24 h before the blood meal. The ICR mice were anesthetized with Avertin (0.2 mL/10 g) via intraperitoneal injection. Then, females were fed on mouse for 15–30 min to initiate reproduction. Three days after the blood meal, the moist filter paper for females to oviposit was placed into the cage. The procedures and experimental protocols applied to mosquitoes and mice were approved by the Committee on the Ethics of Animal Experiments of the National Taiwan University College of Medicine AAALAC-accredited facility (IACUC approval no.: 20200210).

### Cloning and sequencing

The cDNA sequence coding for the *AaEcR* gene was identified from the VectorBase database via the tBLASTn function using the *Aedes albopictus EcR* protein sequence as a template. Full-length *AaEcR* cDNA from the cDNA pool of *Ae*. *aegypti* was amplified with Polymerase Chain Reaction (PCR) using gene-specific primers. The primers used for RNA interference were designed with the E-RNAi tool (Horn and Boutros, 2010). The PCR products were inserted into the pCR2.1-TOPO vector according to the manufacturer’s instructions (ABI/Invitrogen, Carlsbad, CA, USA). Full-length cDNAs and deduced amino acid sequences of *AaEcR* were compared using the Basic Local Alignment Search Tool (BLAST) tool provided by the National Center for Biotechnology Information (NCBI) database and a multiple sequence alignment was performed using the Clustal algorithm [28, 29].

### Viral infection and RNA interference in mosquitoes

Female mosquitoes 3–5 days post-eclosion were thoracically injected with 0.2 μL of biological materials using a Nanoject II AutoNanoliter Injector (Drummond Scientific Company, Broomall, PA, USA). For viral infection, the dengue virus serotype 2 strain 2015TW (DENV2 2015TW; GenBank: KU365901.1), obtained from the Taiwan CDC and amplified in *Ae. aegypti* ATC10 cells, was used (2 × 10^3^ FFU/mosquito); for gene silencing, double-stranded RNA (dsRNA) was generated using in vitro transcription and diluted to a final concentration of 6 μg per μL. Control mosquitoes were injected with ds*LacZ* while experimental mosquitoes were injected with the target gene dsRNA. Knockdown efficiency was confirmed through western blot analysis and quantitative PCR upon harvesting the total protein and RNA from mosquitoes 3 days post-injection [30–32].

### RNA isolation, reverse-transcription, and quantitative real-time PCR (qPCR)

Total RNA from whole mosquitoes, mosquito tissues, or cells was isolated using RNAzol (MRC, Cincinnati, OH, USA) following the manufacturers’ recommendations, and reverse-transcribed with either a random primer or an oligo dT primer to synthesize the cDNA pool (High-Capacity cDNA Reverse Transcription Kits; ABI/Invitrogen, Waltham, MA, USA). Quantitative real-time PCR (qPCR) for the detection of viral RNA or mosquito mRNA expression levels was performed using the SensiFASTTM SYBR Low-ROX Kit (Bioline, Memphis, TN, USA) according to the manufacturers’ instructions and was subsequently analyzed using the QuantStudio 5 Real-Time PCR System (Thermo Fisher Scientific, Waltham, MA, USA). The reactions took place in 96-well plates, where the cDNA of the *S7* ribosomal gene was used as an internal control to normalize the results, and the expression levels of the target genes were determined using gene-specific primers as listed in S1 Table. For each experiment, data were generated from more than three biological cohorts of mosquitoes. Quantitative measurements were conducted in triplicate and normalized against *S7* ribosomal mRNA expression. A fold-change value was derived using the delta-delta Ct method.

### Protein extraction and western blot analysis

Total protein was obtained from homogenized mosquitoes in lysis buffer (50 mM Tris, pH 7.4; 1% IGEPAL; 0.25% sodium deoxycholate; 150 mM NaCl; 1 mM EDTA; 1 mM phenylmethyl-sulfonylfluoride; 1X protease inhibitor mixture; 1X phosphatase inhibitor mixture) (Sigma–Aldrich, St. Louis, MO, USA) and centrifuged at 13,000 rpm at 4 ˚C for 15 min. The supernatant was transferred to a QIAshredder column (QIAGEN, Venlo, Netherlands) and centrifuged again to homogenize the lysates. The flow-through was transferred to new 1.5 mL microcentrifuge tubes and quantified by Bio-Rad Protein assay dye (Bio-Rad, Hercules, CA, USA). The samples were then subjected to western blot analysis using anti-*EcR* (15G1a, Developmental Studies Hybridoma Bank, IA, USA), anti-GAPDH (GTX-100118; Genetex), anti-NS1 (YH0023; Yao-Hong Biotechnology, Taipei, Taiwan), anti-E (YH0026; Yao-Hong Biotechnology, Taipei, Taiwan), anti-phospho-S6K (46121974, Sigma), anti-S6K (SC-8418, Santa Cruz), and anti-mosquito Vg (AaVg) antibodies. The anti-AaVg antibody was a gift from Prof. Alexander Raikhel at the University of California, Riverside, USA [33, 34].

### Focus forming assay

ATC10 cells derived from *Ae. aegypti* were seeded on 96-well culture plates and left adhered at 28 ℃ for 1 day before sample harvesting. Samples were harvested separately in 100 μL of serum-free medium and homogenized, followed by centrifugation. After discarding the pellet, the supernatant was moved to a new Eppendorf vial and serially diluted tenfold; 50 μL of the diluted samples were added to the pre-seeded cells and left at 28 ℃ for 2 h. Cells were then treated with 1% methylcellulose insect cell medium with 1% FBS per well. The virus was incubated with cells at 28 °C for 4 days to allow for further infection. After 4 days, 4% paraformaldehyde was added to each well and fixed for 30 min at 4 °C, followed by washing and permeabilizing with 0.1% Triton X-100 at room temperature (RT) for 1 h. The cells were next blocked with 0.5X PAT at RT for 1 h. The 0.5X PAT was then discarded and replaced with 30 μL of mouse monoclonal anti-DENV2 NS1 (1:1000, Yao-Hong Biotechnology) per well and the plate was left overnight at 4 °C. The plate was then subjected to washing with PBS four times, then each well was incubated with 30 μL of fluorescent-conjugated goat anti-mouse antibody (1:500, Invitrogen) at RT for 2 h. Following this incubation, the cells were washed and mounted with mounting medium containing DAPI. Fluorescent images of each plate were then captured using the High-Content Imaging System (Molecular Devices, San Jose, CA, USA). The viral foci were counted using ImageJ software and the titer was determined by multiplying the dilution factors. Viral titer was recorded in units of focus forming units per mL (FFU/mL) [35, 36].

### Oviposition and hatching assay

For the oviposition assay, blood-fed female mosquitoes were individually separated into 50 mL centrifuge tubes containing wet filter paper wrapped around a water-soaked cotton ball, and 3–5 days later, the filter papers, now with eggs attached, were air-dried and the number of eggs was counted under a microscope. For the hatching assay, the filter papers with the eggs were placed in a container with distilled water and exposed under vacuum conditions for 1 h. The number of hatched larvae was then calculated [29, 30].

### Statistical analysis

All values are presented as means ± standard deviation (SD) or ± standard error of the mean (SEM), with experiments being performed a minimum of three times. Differences of the values between the two groups were analyzed using the unpaired nonparametric Mann–Whitney *U* test. Differences of the proportion between two groups were analyzed using Fisher’s exact test. A one-way analysis of variance (ANOVA), followed by a post hoc Tukey’s honestly significant difference (HSD) test, was conducted to compare multiple treatment groups. In all statistical comparisons, a *P* < 0.05 was defined as significant. Graphpad Prism 8.0 software was used for all calculations.

## Results

### AaEcR knockdown suppresses viral genome replication, protein synthesis, and virion production in Aedes aegypti

**Fig. 1:**
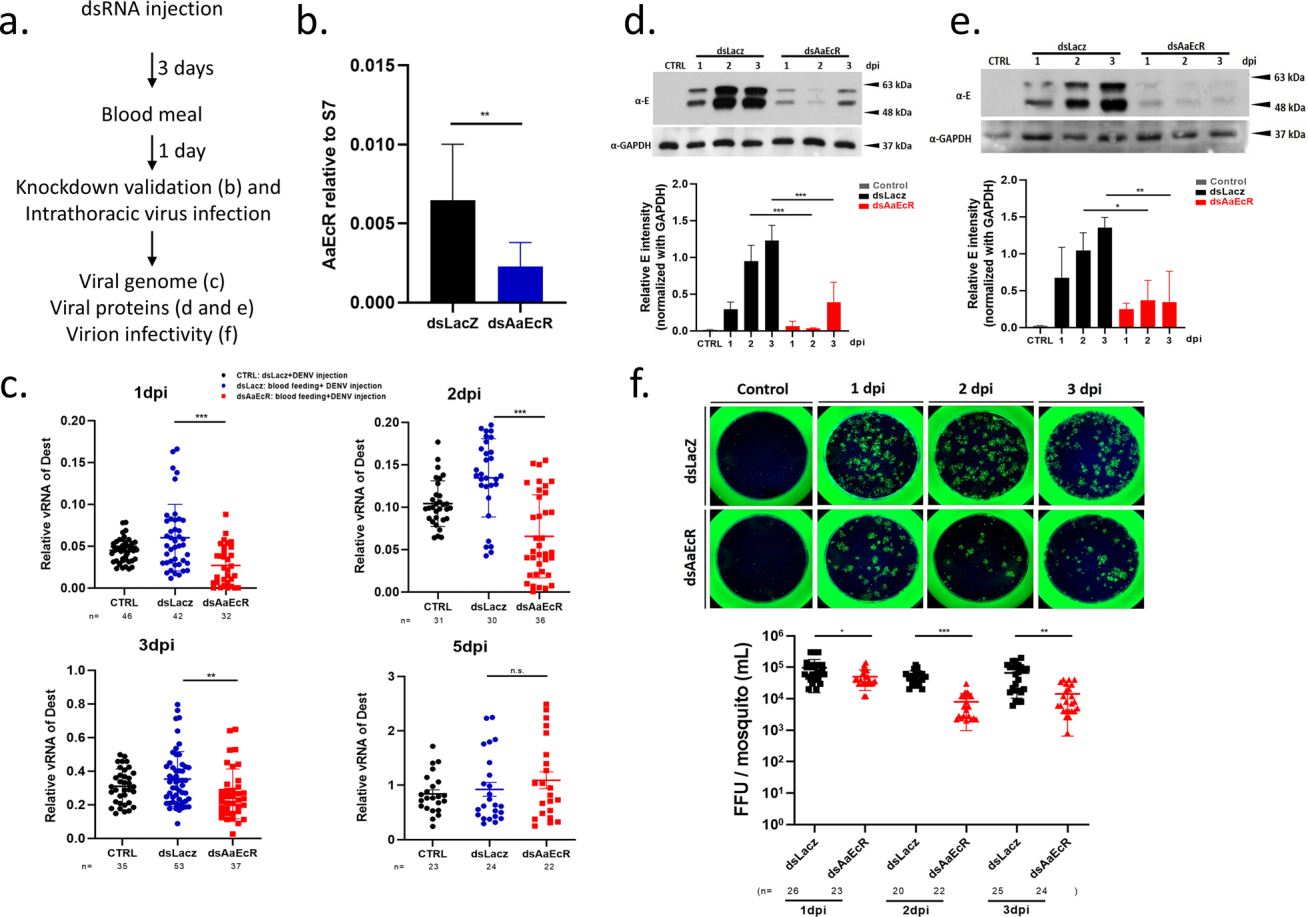
Effect of *AaEcR* on DENV replication. **a** Schematic of study design. Wild-type female mosquitoes were injected with either ds*LacZ* or ds*AaEcR* 3 days before mouse blood feeding, followed by thoracic injection of DENV2 2015TW 1 day post-blood meal, with samples collected for the following experiments. **b** Mosquitoes were collected 1 day following the blood meal for RNA extraction and qPCR analysis to validate ds*AaEcR* knockdown efficiency. The ds*LacZ* group served as the control for dsRNA-mediated gene silencing. **c** After dsRNA injection, all groups, except the control, were fed with mouse blood. The dengue virus genome was quantified using qPCR with DENV2-specific primers, and results were normalized against ribosomal *S7* at 1, 2, 3, and 5 days post-infection (dpi). Data were analyzed using the Mann–Whitney *U* test (n.s., not significant; **P < 0.01, ***P < 0.001); *n* represents the number of samples. **d** Fat bodies collected from 20 to 30 mosquitoes were pooled into lysis buffer at 1, 2, and 3 dpi. Tissue proteins were extracted and subjected to immunoblotting with α-E protein antibody, using anti-Glyceraldehyde 3-phosphate dehydrogenase antibody (α-GAPDH) as the internal control. The bar chart, created using ImageJ, quantifies the viral envelope protein from the fat body. Data were analyzed using the Mann–Whitney *U* test (***P < 0.001). **e** Midgut proteins collected from pooled individuals at 1, 2, and 3 dpi were examined by immunoblotting using α-E protein antibody. The viral envelope proteins from midguts were quantified and normalized to GAPDH using ImageJ. Data were analyzed using the Mann–Whitney *U* test (*P < 0.05, **P < 0.01). **f** Whole mosquitoes were collected into serum-free medium at 1, 2, and 3 dpi. The focus forming assay with α-NS1 monoclonal antibody (green) and DAPI (blue) was used to localize the virus particles and the nuclei, respectively. Viral titer was determined by counting the number of virus particles, with each point representing a single mosquito. Dot plots are presented as mean ± SEM from three independent experiments, analyzed using the Mann–Whitney U test (*P < 0.05, **P < 0.01, ***P < 0.001)

While the role of the 20E signaling pathway in mosquito reproduction is well established, its involvement in immunity is less clear [37, 38]. A previous study by Mao et al. reported that *EcR* knockdown in *Aedes aegypti* led to the upregulation of components of the IMD pathway and enhanced resistance to Gram-negative bacterial infections [39]. On the basis of these findings, we hypothesized that *AaEcR* might similarly influence antiviral responses and impact dengue virus (DENV2) replication in mosquitoes. To test this, we silenced *AaEcR* expression using dsRNA and assessed its effect on viral replication, viral protein production, and virion infectivity. Female mosquitoes, aged 3–5 days, were injected with either ds*AaEcR* or ds*LacZ*. To induce *AaEcR* expression, blood feeding was performed 1 day prior to virus exposure. Mosquitoes were then infected with the DENV2 2015TW strain through thoracic microinjection (Fig. 1a). Knockdown efficiency was confirmed via qPCR (Fig. 1b). Quantification of viral RNA showed that *AaEcR* silencing significantly reduced DENV2 replication at 1, 2, and 3 days post-infection (dpi) compared with controls, although no significant difference was observed at 5 dpi (Fig. 1c). These results indicate that *AaEcR* plays a role in supporting DENV2 infection following injection challenge.

We next examined DENV2 E protein levels in the fat body and midgut after thoracic injection of the virus. Immunoblotting revealed a marked d*EcR* ease in E protein expression in both tissues at 2 and 3 dpi in ds*AaEcR*-injected mosquitoes compared with ds*LacZ* controls (Fig. 1d, e). Densitometric quantification confirmed significant reductions in E protein levels in both the fat body (Fig. 1d) and midgut (Fig. 1e), suggesting that *AaEcR* is required for efficient viral protein synthesis in these tissues. To further evaluate the effect of *AaEcR* silencing on DENV2 infectivity, we performed a focus-forming assay (FFA). Mosquitoes were infected via thoracic injection, and viral titers were measured in individual mosquitoes at 1, 2, and 3 dpi. Viral infectivity was significantly reduced in the ds*AaEcR*-injected group compared with controls (Fig. 1f). Quantification of focus-forming units per mosquito confirmed these findings (Fig. 1f). Together, these results demonstrate that *AaEcR* supports DENV2 genome replication, E protein expression, and virion production in *Aedes aegypti*.

### AaEcR knockdown enhances expression of IMD pathway-associated antimicrobial peptides during DENV2 infection

Previous studies have shown that *EcR* can influence pathogen susceptibility by modulating the mosquito immune response [38, 40–42]. In mosquitoes, one of the primary defense mechanisms against pathogens involves the induction of AMPs via innate immune pathways. To investigate whether *AaEcR* modulates immune gene expression during DENV2 infection, we analyzed the expression profiles of selected AMPs and immune-related transcription factors following *AaEcR* knockdown. Female mosquitoes were injected with either ds*AaEcR* or ds*LacZ* 3 days before receiving a blood meal. At 24 hPBM, mosquitoes were infected with DENV2 2015TW via thoracic injection. Total RNA was extracted at 0 (pre-infection), 3, and 5 dpi, and transcript levels of four representative AMPs—*cecropin B* (*CECB*), *diptericin* (*Dpt*), *defensin C* (*DEFC*), and *gambicin* (*GAM*)—as well as the immune pathway transcription factors *Rel1* (Toll pathway) and *Rel2* (IMD pathway), were measured using qPCR.

**Fig. 2:**
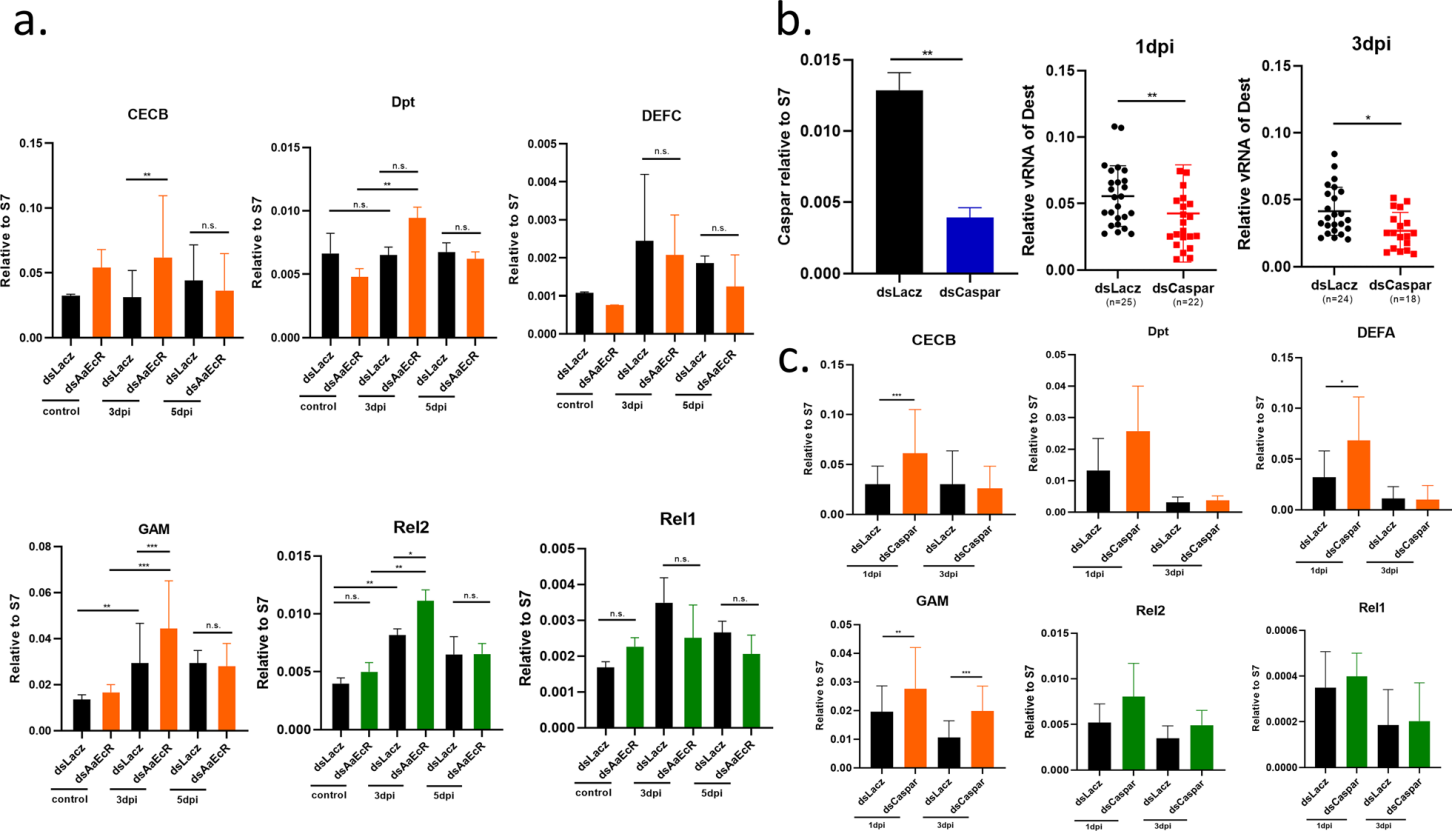
Effect of *AaEcR* on production of AMP. **a** At 24 h post-blood meal (hPBM), ds*LacZ*- and ds*AaEcR*-injected mosquitoes were intrathoracically injected with medium containing dengue virus (experimental group) or with serum-free medium (control group). The transcript levels of various antimicrobial peptides (*CECB*, *Dpt*, *DEFC*, and *GAM*) and transcription factors involved in the mosquito immune pathway (*Rel1* and *Rel2*) were measured by qPCR and normalized with ribosomal *S7* at 3 and 5 days post-infection (dpi). Data were analyzed using the one-way ANOVA (n.s., not significant; *P < 0.05, **P < 0.01, ***P < 0.001). **b** Mosquitoes injected with ds*LacZ* and ds*Caspar* were infected with DENV2 2015TW at 24 hPBM. The mRNA expression level of the viral genome was quantified using qPCR at 1 and 3 dpi. Results were analyzed using the Mann–Whitney *U* test (*P < 0.05, **P < 0.01); *n* represents the number of samples. **c** At 24 hPBM, ds*LacZ*- and ds*Caspar*-injected mosquitoes were infected with DENV2 2015TW. The transcript levels of various antimicrobial peptides (*CECB*, *Dpt*, *DEFA*, and *GAM*) and transcription factors of the mosquito immune pathway (*Rel1* and *Rel2*) were quantified by qPCR and normalized with ribosomal *S7* at 1 and 3 dpi. Data were analyzed using one-way ANOVA (*P < 0.05, **P < 0.01, ***P < 0.001). Results are shown as mean ± SEM from three independent experiments

Among the AMPs analyzed, *GAM* expression was notably upregulated in *AaEcR*-silenced mosquitoes, particularly at 3 dpi (Fig. 2a). In addition, *Rel2* transcript levels were elevated following *AaEcR* knockdown, while *Rel1* expression remained unchanged (Fig. 2a). These results suggest that *AaEcR* potentially facilitates viral replication by suppressing Rel2-mediated AMP responses during infection.

### AaEcR silencing enhances multiple antiviral immune pathways to suppress DENV2 replication

Building on our previous findings that *AaEcR* regulates the IMD pathway and modulates AMP expression, we further investigated whether the IMD pathway directly influences dengue virus replication through AMP regulation. To assess this, we silenced *Caspar*, a known negative regulator of the IMD pathway [43], using dsRNA. Female mosquitoes were injected with either ds*Caspar* or ds*LacZ* 3 days prior to a blood meal and subsequently infected with DENV2 2015TW via thoracic injection 24 hPBM. Compared with the ds*LacZ*-injected controls, *Caspar* silencing significantly reduced viral RNA levels at 1 and 3 dpi (Fig. 2b). This indicates that activation of the IMD pathway impairs dengue virus replication. To determine whether this antiviral effect was associated with AMP induction, we quantified the transcript levels of several immune-related genes, including *cecropin B* (*CECB*), *diptericin* (*Dpt*), *defensin A* (*DEFA*), and *GAM*, along with the transcription factors *Rel1* and *Rel2*. *Caspar* knockdown led to elevated expression of AMPs (*CECB*, *DEFA*, and *GAM*) and *Rel2*, but not *Rel1* (Fig. 2c). These results echo the role of *Caspar* as a well-known negative regulator of the IMD pathway, suggesting that the IMD pathway specifically regulates AMP expression to limit viral replication. Collectively, these findings support the conclusion that *AaEcR* promotes DENV2 replication by suppressing IMD pathway activity, and that its silencing reduces viral burden through upregulation of AMPs via *Rel2*-mediated signaling.

**Fig. 3:**
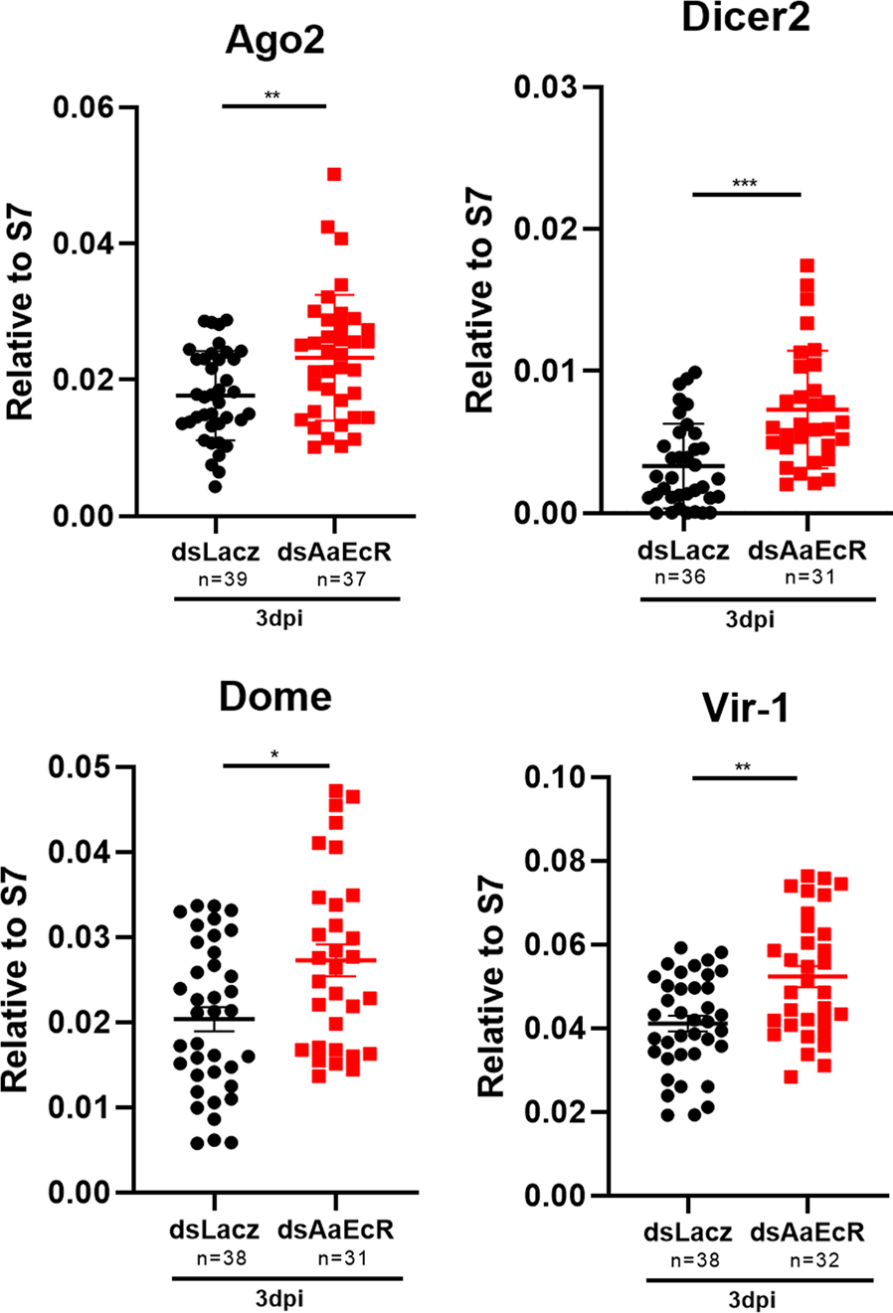
Effect of *AaEcR* on the RNAi and JAK-STAT pathways. Wild-type female mosquitoes were injected with either ds*LacZ* or ds*AaEcR* 3 days before feeding on mouse blood. At 24 h post-blood meal, mosquitoes were intrathoracically infected with DENV2 2015TW. At 3 days post-infection, whole mosquitoes were placed into RNAzol. The extracted RNA was converted to cDNA through reverse transcription. The mRNA expression levels of Argonaute 2 (*AGO2*), *Dicer2*, *Dome*, and virus-induced RNA 1 (*Vir-1*) were quantified using qPCR and normalized with ribosomal *S7*. The data were analyzed using one-way ANOVA (*P < 0.05, **P < 0.01, ***P < 0.001). Results are presented as mean ± SEM from three independent experiments

In mosquitoes, antiviral immunity involves the coordinated activity of several innate immune pathways, including the Toll, IMD, RNAi, and JAK-STAT pathways. To investigate whether *AaEcR* modulates additional antiviral responses beyond the IMD pathway, we examined the expression of key components from the RNAi and JAK-STAT pathways following *AaEcR* silencing (Fig. 3). Female mosquitoes were injected with either ds*AaEcR* or ds*LacZ* and infected with DENV2 2015TW either via thoracic DENV2 infection. Whole mosquito RNA was extracted for qPCR analysis. Transcript levels of *Argonaute 2* (*Ago2*) and *Dicer2*, two critical components of the RNAi pathway, were significantly elevated in *AaEcR*-silenced mosquitoes compared with controls, suggesting that RNAi activity was enhanced upon *AaEcR* knockdown (Fig. 3).

To further test our hypothesis that *AaEcR* modulates multiple immune pathways, we assessed the expression of virus-induced RNA-1 (*Vir-1*) and *Dome*, key components of the JAK-STAT pathway. Following either thoracic DENV2 infection (Fig. 3), both *Vir-1* and *Dome* transcript levels were significantly increased in *AaEcR*-silenced mosquitoes relative to controls. Together, these results suggest that *AaEcR* knockdown not only activates the IMD pathway, but also enhances the RNAi and JAK-STAT pathways, contributing to broad-spectrum antiviral immunity and reduced dengue virus replication in *Aedes aegypti*.

### AaEcR knockdown impairs ovarian development and reduces fecundity in *Aedes aegypti*

Given the known role of *EcR* in insect reproduction, we examined *AaEcR* expression dynamics in *Aedes aegypti* females. Quantitative PCR (qPCR) (Supplementary Fig. 1a) and immunoblotting (Supplementary Fig. 1b) were performed on fat body, midgut, and ovary tissues collected at the pre-vitellogenic (PV) stage and at 6, 12, 24, 48, and 72 hPBM. *AaEcR* expression was upregulated in all three tissues following a blood meal, with the most pronounced increase observed in fat body tissue, peaking at 24 hPBM. These results, together with *EcR*’s established role in reproduction in other insect species, suggest that *AaEcR* plays a regulatory role during vitellogenesis in *Aedes aegypti*.

**Fig. 4:**
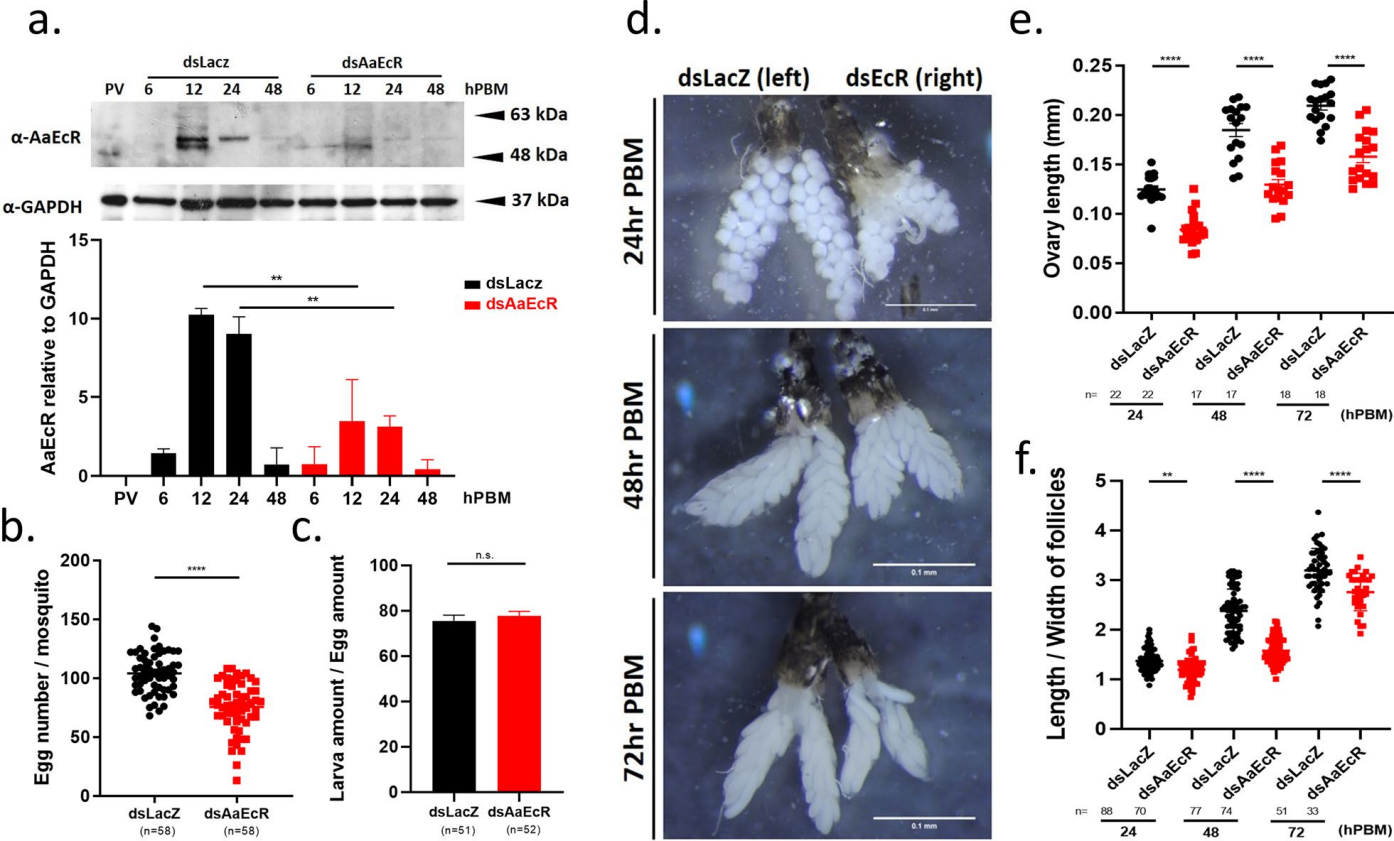
Effect of *AaEcR* on egg production. Wild-type female mosquitoes (3–5 days old) were intrathoracically injected with double-stranded RNA targeting either *LacZ* (ds*LacZ*) or *AaEcR* (ds*AaEcR*), and 3 days post-injection, mosquitoes were fed on mouse blood. (**a**, upper) Protein lysates from mosquito fat bodies were collected at various time points PBM and subjected to immunoblotting using a monoclonal α*-Drosophila* EcR antibody. (**b**, lower) Densitometric quantification of *AaEcR* protein levels was performed using ImageJ software and normalized to GAPDH as a loading control. Data represent mean ± SEM from three independent experiments. Statistical significance was assessed using the Mann–Whitney *U* test (**P < 0.01). **c** Female wild-type mosquitoes were injected with either ds*LacZ* or ds*AaEcR* 3 days prior to feeding on mouse blood. At 72 h post-blood meal (hPBM), blood-fed mosquitoes were transferred to oviposition tubes and then given another 72 h to oviposit on filter paper. The number of eggs laid by each mosquito was counted under a microscope. **d** The number of hatched larvae were calculated and normalized against the number of eggs. The data were analyzed using the Mann–Whitney *U* test (n.s., not significant; ****P < 0.0001). **e** Ovaries from ds*LacZ*- and ds*AaEcR*-injected mosquitoes were dissected at 24, 48, and 72 hPBM. Representative images were captured under a microscope. **f** The lengths of ovaries and **g** the length-to-width ratios of follicles were measured in ImageJ. A scale bar of 0.1 mm is provided for reference. Data were analyzed using the Mann–Whitney *U* test (**P < 0.01, ****P < 0.0001). Dot plots and bar plots are shown as mean ± SEM from three independent experiments; *n* represents the number of samples

To investigate the functional role of *AaEcR* in mosquito reproduction, RNAi was used to silence *AaEcR* expression. Female mosquitoes (3–5 days post-eclosion) were injected with double-stranded RNA (dsRNA) targeting *AaEcR* (ds*AaEcR*) or with control dsRNA targeting *LacZ* (ds*LacZ*), and 3 days post-injection, *AaEcR* knockdown efficiency was confirmed by immunoblotting using an anti-Drosophila EcR antibody (Fig. 4a). This revealed a ~50% reduction in protein levels (Fig. 4a), indicating effective gene silencing.

To assess the impact of *AaEcR* knockdown on reproduction, dsRNA-injected females were blood-fed to initiate vitellogenesis. Compared with controls, ds*AaEcR*-injected mosquitoes laid ~30% fewer eggs (an average of ~70 eggs versus ~100 eggs per female; Fig. 4b). However, egg viability was unaffected, with both groups showing similar hatching rates (~80%; Fig. 4c). These results suggest that *AaEcR* knockdown primarily impairs fecundity without significantly affecting fertility.

To determine whether the reduction in egg production was due to impaired ovarian development, ovaries were dissected and analyzed at multiple timepoints post-blood meal. Compared with controls, *AaEcR*-silenced mosquitoes exhibited severely underdeveloped ovaries (Fig. 4d), characterized by significantly shorter ovary lengths (Fig. 4e) and smaller follicle sizes (Fig. 4f). Together, these findings indicate that *AaEcR* is critical for ovarian development and plays a key role in regulating fecundity in *Aedes aegypti*.

### AaEcR knockdown suppresses vitellogenin expression at both transcriptional and translational levels

**Fig. 5:**
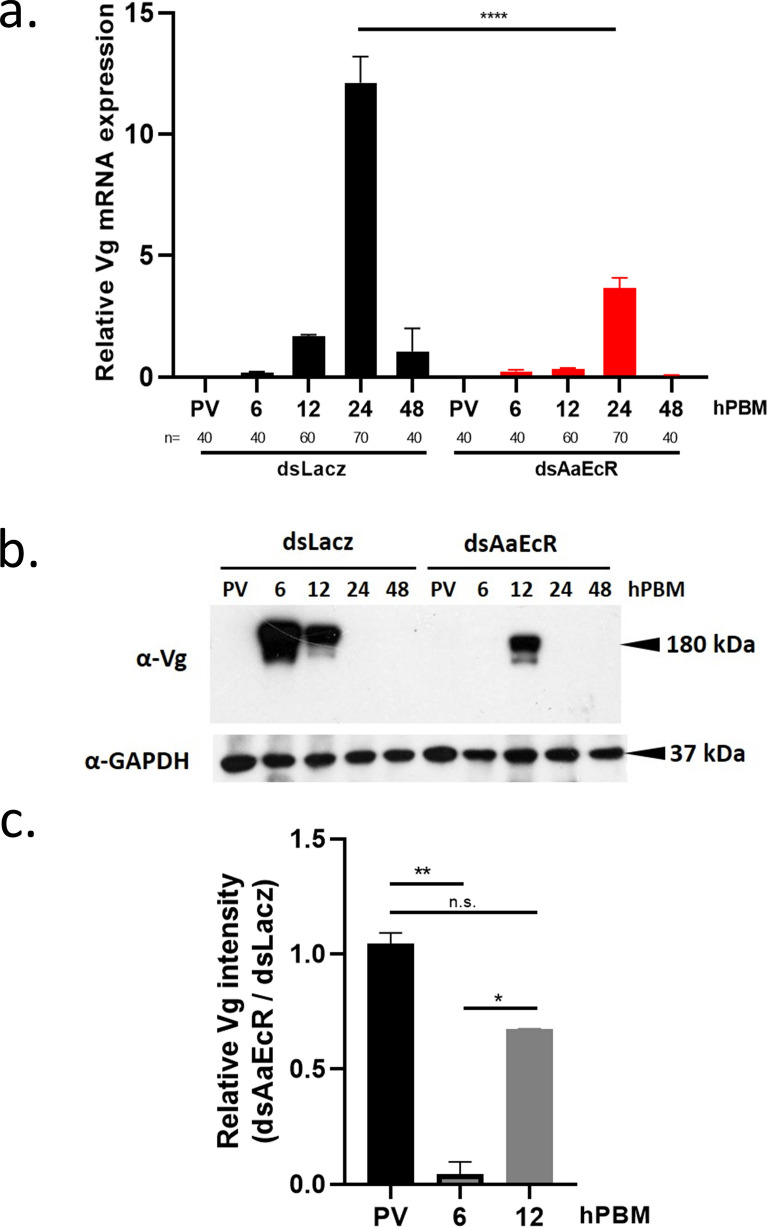
Knockdown of *AaEcR* inhibits vitellogenin (Vg) expression. **a** Wild-type female mosquitoes were injected with dsRNA 3 days before feeding on mouse blood. Total RNA from the fat body of ds*LacZ*- and ds*AaEcR*-injected mosquitoes was collected and extracted at the pre-vitellogenic (PV) stage and 6, 12, 24, 48, and 72 h post-blood meal (hPBM). The mRNA expression of vitellogenin (Vg) was determined by qPCR, with ribosomal S7 serving as an internal control to normalize the results. The data were analyzed using the Mann–Whitney *U* test (****P < 0.0001); *n* represents the number of samples. **b** At the PV stage and 6, 12, 24, 48, and 72 hPBM, protein from the fat body of ds*LacZ*- and ds*AaEcR*-injected mosquitoes was collected and isolated for immunoblotting with the α-Vg antibody. **c** The relative intensity of the Vg protein was quantified using ImageJ and normalized to the ratio of the ds*AaEcR* group to the ds*LacZ* group. The data were analyzed using the Mann–Whitney *U* test (n.s., not significant; *P < 0.05, **P < 0.01). Bar plots are shown as mean ± SEM from three independent experiments

Vitellogenin (Vg), the major yolk protein precursor, is essential for egg formation and is known to be activated by ecdysone signaling. Previous studies have shown that Vg gene expression is regulated both directly and indirectly by *EcR *[21–24]. To elucidate the role of *AaEcR* in Vg regulation, we examined Vg expression in the fat bodies of *Aedes aegypti* females following *AaEcR* knockdown. Total RNA and protein were extracted from the fat bodies of ds*LacZ*- and ds*AaEcR*-injected mosquitoes at various timepoints post-blood meal. qPCR revealed a marked (~70%) reduction in Vg mRNA levels in the *AaEcR* knockdown group compared with controls at 24 hPBM (Fig. 5a). Consistent with the transcriptional data, immunoblotting with an anti-Vg antibody (∼180 kDa) showed significantly reduced Vg protein levels at 6 and 12 hPBM in *AaEcR*-silenced mosquitoes (Fig. 5b), with quantification presented in Fig. 5C. These findings indicate that *AaEcR* is required for proper *Vg* expression at both the transcriptional and translational levels. The suppression of *Vg* expression in *AaEcR*-silenced mosquitoes likely contributes to impaired ovarian development and reduced fecundity.

### AaEcR knockdown reduces TOR pathway activation in the fat body

**Fig. 6:**
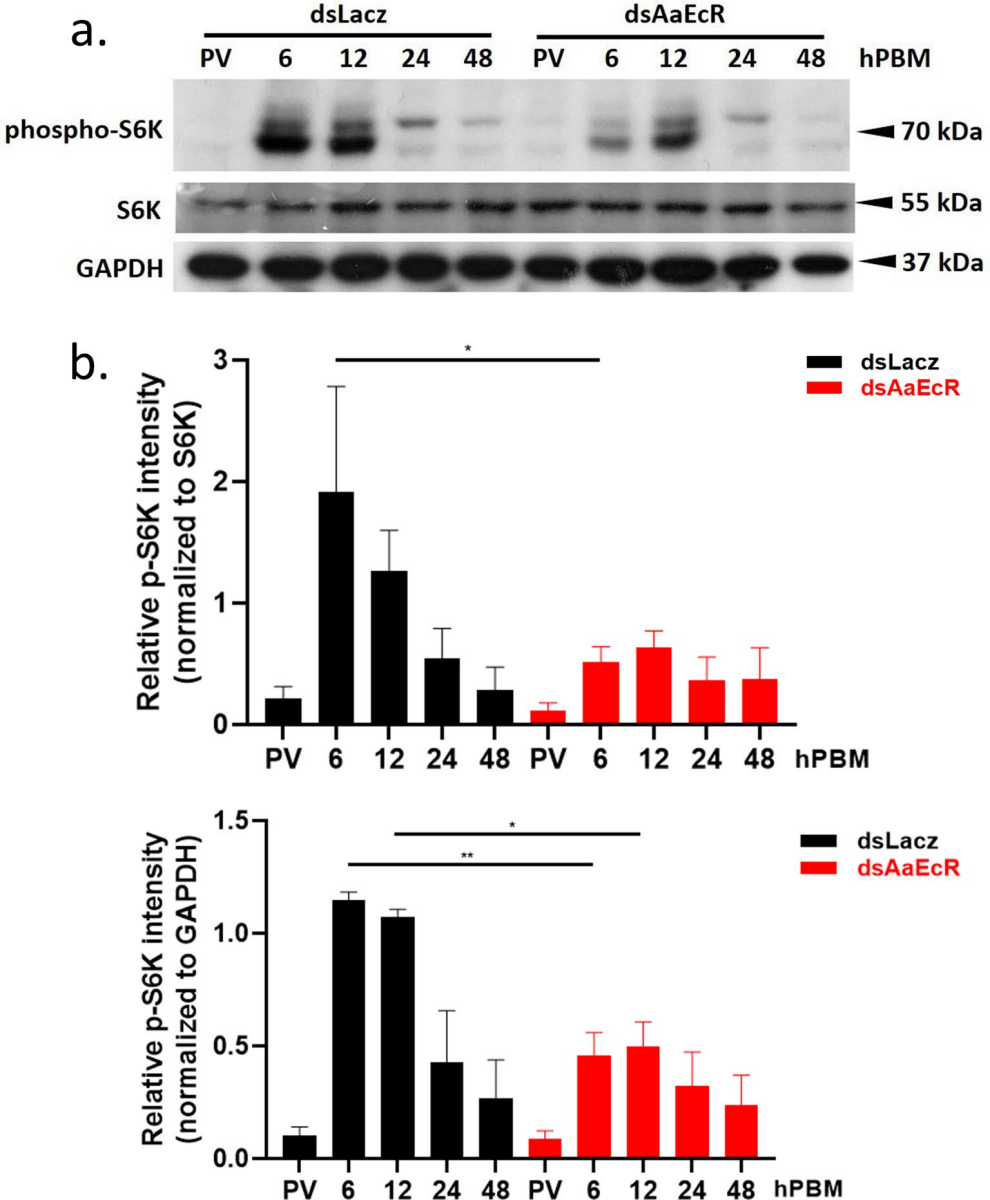
Effect of AaEcR on the TOR signaling pathway. Wild-type female mosquitoes were intrathoracically injected with either ds*LacZ* or ds*AaEcR* 3 days before feeding on mouse blood. Dissections of the fat body were performed at pre-vitellogenic (PV) stage and 6, 12, 24, and 48 h post-blood meal. **a** Protein from the fat body of ds*LacZ*- and ds*AaEcR*-injected mosquitoes was collected and isolated for immunoblotting with the α-phospho-S6K antibody. **b** Quantification of phospho-S6K relative intensity was performed using ImageJ and normalized against GAPDH. Data were analyzed using the Mann–Whitney *U* test (n.s., not significant; *P < 0.05, **P < 0.01, ****P < 0.0001). Bar plots are shown as mean ± SEM from three independent experiments

Previous studies have demonstrated that Vg expression is regulated by the TOR signaling pathway, with phosphorylation of S6 kinase (S6K) serving as a key indicator of TOR pathway activation [44, 45]. To determine whether *AaEcR* knockdown influences TOR signaling, we measured levels of phosphorylated S6K (phospho-S6K) in mosquito fat bodies following blood feeding. Fat bodies were dissected from ds*LacZ*- and ds*AaEcR*-injected females at multiple timepoints post-blood meal, and total protein was extracted for immunoblotting. Using an anti-phospho-p70 S6K antibody targeting ~70 kDa proteins, we observed a significant reduction in phospho-S6K levels in *AaEcR*-silenced mosquitoes at 6 and 12 hPBM compared with controls (Fig. 6a). The densitometric quantification of the phospho-S6K signal is shown in Fig. 6b. These results suggest that *AaEcR* regulates Vg protein expression through the TOR–S6K pathway. The observed decrease in phosphorylated S6K following *AaEcR* knockdown likely contributes to the reduction in Vg expression, impaired ovarian development, and reduced reproductive output.

## Discussion

The *EcR* is a master regulator in the life cycle of *Aedes aegypti*, integrating hormonal signals to control development, metamorphosis, and reproduction [37, 46–50]. Its central role in these processes makes it a key focus for research aimed at controlling mosquito populations and limiting the spread of mosquito-borne diseases. Here, we establish *AaEcR* as a central regulator of both viral susceptibility and mosquito reproduction, with implications for vectorial capacity and mosquito physiology. Our findings show that *AaEcR* is highly conserved among dipteran species, robustly upregulated following a blood meal, and plays dual roles in suppressing innate antiviral immunity and promoting vitellogenesis.

*AaEcR* emerged as a critical modulator of DENV2 infection. Silencing *AaEcR* significantly suppressed DENV2 genome replication, E protein expression, and virion production following intrathoracic injection of DENV2 (Fig. 1). These effects appeared early in infection and were associated with increased expression of AMPs, particularly *GAM*, as well as elevated levels of *Rel2*, a key transcription factor in the IMD pathway (Fig. 2). These results suggest that *AaEcR* facilitates viral replication, at least in part, by suppressing immune responses mediated by the IMD pathway.

This immunosuppressive role of *EcR* was further substantiated by Caspar knockdown experiments, where *Caspar* knockdown activated the IMD pathway and led to reduced viral loads (Fig. 2). Together, these findings suggest that *AaEcR* functions as a negative regulator of IMD-mediated antiviral responses, creating a favorable intracellular environment for DENV2 replication during the reproductive cycle. Beyond the IMD pathway, our data indicate that *AaEcR* modulates multiple components of mosquito innate immunity. *AaEcR* knockdown led to the upregulation of RNAi pathway genes (*Ago2*, *Dicer2*) and JAK-STAT pathway components (*Vir-1*, *Dome*), indicating *AaEcR* has broad immunosuppressive effects (Fig. 3). These findings are particularly intriguing in the context of blood meal-triggered hormonal shifts, as they suggest that 20E signaling via *AaEcR* may temporarily dampen antiviral defenses to prioritize reproductive processes. This tradeoff may inadvertently facilitate arboviral transmission by enhancing midgut and systemic infection during the vitellogenic phase.

Since AaEcR is a blood-fed induced gene, mosquitoes received a blood meal 1 day prior to DENV infection to enable investigation of its role in DENV2 replication. Consequently, most DENV2 infection experiments in this study utilized intrathoracic injection rather than natural oral infection to eliminate the confounding effects of multiple blood feedings on viral infection. Intrathoracic injections serve as an effective method for evaluating viral replication and systemic antiviral responses. However, it does bypass several critical steps of natural dengue virus transmission, which constitutes a limitation of this study.

The reproductive functions of *EcR* in insects are well documented, particularly in regulating Vg synthesis in response to 20E signaling [21–24]. In *Aedes aegypti*, our results demonstrate that *AaEcR* is strongly upregulated in reproductive tissues post-blood meal, particularly in the fat body, a major site of yolk protein synthesis (Supplementary Fig. 1). Functional knockdown of *AaEcR* significantly impaired ovarian development, reduced follicle size and number, and led to a ~30% decrease in egg production, without affecting egg viability (Fig. 4). This reinforces the conserved role of *EcR* in coordinating reproductive maturation and highlights its essential function during vitellogenesis in mosquitoes.

At the molecular level, *AaEcR* knockdown resulted in the marked downregulation of *Vg* transcription and reduced Vg protein levels, demonstrating that *AaEcR* is required for *Vg* production at both the transcriptional and translational levels (Fig. 5). Interestingly, we observed that TOR signaling activity, as indicated by phospho-S6K levels, was significantly reduced in *AaEcR*-depleted fat bodies (Fig. 6). This suggests that *EcR*-mediated activation of the TOR pathway may act synergistically with ecdysone signaling to enhance yolk protein production. Given the established crosstalk between hormonal and nutrient-sensing pathways during egg maturation, our findings provide mechanistic insight into how ecdysone signaling interfaces with TOR to drive reproductive output in *Aedes aegypti*.

Taken together, our results highlight the dual roles of *AaEcR* in *Aedes aegypti*: acting as a positive regulator of fecundity through the TOR-vitellogenin axis while concurrently dampening antiviral immunity by repressing the IMD, RNAi, and JAK-STAT pathways. This dual function suggests that targeting *AaEcR* could be a powerful strategy for both population suppression and transmission control. RNAi-based approaches or small-molecule inhibitors that disrupt *AaEcR* signaling may reduce egg production while enhancing the mosquito’s natural antiviral defenses, thereby lowering their competence as a vector of dengue and potentially other arboviruses.

The dual roles of *EcR* in both immunity and reproduction in *Ae. aegypti* indicate potential interactions between these biological pathways. Previous studies have established that yolk protein synthesis influences pathogen infection [51–57], while additional research has demonstrated that 20E affects *Plasmodium* development in *Anopheles *[38, 58]. However, further investigation is necessary to clarify the specific mechanisms linking immunity and reproductive processes [59–62].

Our study identifies *AaEcR* as a pivotal molecular node linking reproductive physiology and vector immunity. The opposing effects of *AaEcR* on fecundity and antiviral resistance highlight the existence of an evolutionary tradeoff between reproduction and immunity in mosquitoes and offer a promising target for integrated vector management strategies.

## Conclusions

In this study, we establish the *Aedes aegypti* ecdysone receptor (*AaEcR*) as a dual regulator of mosquito physiology and vector competence. Functional silencing of *AaEcR* suppressed dengue virus genome replication, protein expression, and virion production by enhancing multiple innate immune pathways, including IMD, RNAi, and JAK-STAT signaling, and by upregulating antimicrobial peptide expression. At the same time, *AaEcR* knockdown impaired ovarian development and reduced follicle number and size, and d*EcR* eased egg production by approximately 30% through disruption of TOR signaling pathway. These findings reveal a mechanistic link between hormonal regulation, immunity, and fecundity, highlighting an evolutionary tradeoff where reproduction is prioritized at the expense of antiviral defense. By defining *AaEcR* as a molecular hub that governs both reproductive fitness and viral susceptibility, our study provides a strong conceptual and translational basis for targeting steroid hormone signaling as an integrated vector control strategy that could simultaneously reduce mosquito populations and limit arbovirus transmission.

## Supplementary Information

Additional file 1.

Additional file 2. Figure 1. Temporal expression profile of *AaEcR* mRNA and protein in selected tissues of wild-type *Aedes aegypti* following a blood meal. Wild-type female mosquitoes (3–5 days old) were starved for 24 h prior to receiving a blood meal. Tissues including the fat body, midgut, and ovary were dissected at defined timepoints: pre-vitellogenic stage (PV), and at 6, 12, 24, 48, and 72 h post-blood meal (hPBM). **a** Total RNA was extracted from these tissues and reverse-transcribed into cDNA. Quantitative PCR (qPCR) was performed to assess *AaEcR* mRNA expression levels. Ribosomal protein *S7* was used as the internal control for normalization. The data were analyzed by the Mann–Whitney *U* test (***P<0.001, ****P<0.0001). **b** Protein lysates from the fat body, midgut, and ovary were collected at the same timepoints and subjected to immunoblotting using a monoclonal α-*AaEcR* antibody

## Data Availability

All data generated or analyzed during this study are included in this published article.

## Abbreviations

DENV: Dengue virus
EcR: Ecdysone receptor
AMPs: Antimicrobial peptides
IMD: Immune deficiency
RNAi: RNA interference
TOR: Target of rapamycin
YPPs: Yolk protein precursors
Vg: Vitellogenin
20E: 20-Hydroxyecdysone
PBM: Post-blood meal
