# Supplementary material for: Dual functions of the Aedes aegypti ecdysone receptor in dengue virus replication and reproduction control
Source: Parasit Vectors. 2026 Mar 22;19:190. doi: 10.1186/s13071-026-07298-0 (PMC13130442; doi:10.1186/s13071-026-07298-0)
Supplement: Supplementary file 1 — Additional file 1. [file 13071_2026_7298_MOESM1_ESM.pdf]

S1 Table. Oligonucleotides used in this study

OligoNT	Sequence (5'– 3')	Usage
S7 CHK_F	TCAGTGTACAAGAAGCTGACCGGA	Quantification of mRNA expression via qPCR
S7 CHK_R	TTCCGCGCGCTCACTTATTAGATT	
Dest1_F	GAAGACATTGACTGYTGGTGCAA	
Dest1_R	CGATGTTTCCACGCCCCTTC	
AaEcR CHK_F	GCCCTCAATGCTACCACGAT	
AaEcR CHK_R	GGAAGGGGTTCCACCTTGAC	
AaCaspar CHK_F	GAATCCGAGCGAGCCGATGC	
AaCaspar CHK_R	CGTAGTCCAGCGTTGTGAGGTC	
AaVg CHK_F	ATGCACCGTCTGCCATC	
AaVg CHK_R	GTTCGTAGTTGGAAAGCTCG7	
CECB CHK_F	AAGCTGGTCGGCTGAAGAAG	
CECB CHK_R	ATCTTCCCAGTCCCTTGATG	
Dpt CHK_F	TGTCCATCCGAGTGAGACGT	
Dpt CHK_R	CTCCCTGAAATCCACCAAAA	

DEFA CHK_F	CTATCAGGCTGCCGTGGAG	
DEFA CHK_R	CAATGAGCAGCACAAGCACTCT C	
DEFC CHK_F	GCCTCAGTGCAATCTTCACA	
DEFC CHK_R	CGTTTCAAGCGGAAGTTTTC	
GAM CHK_F	GTTCCTCCTGCAAGGCATATG	
GAM CHK_R	GACAGTCACTGCAGCTTCTTAT TG	
Rel1 CHK_F	TCCCCACTTTACGGCAACAC	
Rel1 CHK_R	TGGTTCTGCCGGCATTGTA	
Rel2 CHK_F	GGATGCGGTGTCGGTTGGTC	
Rel2 CHK_R	CGTCCTTGGCTTGGCTGTTG	
Ago2 CHK_F	TACCCGGCTCCAACCTATTA	
Ago2 CHK_R	CTGCATTCTCTCGTACTCCTTG	
Dicer2 CHK_F	CACCGACACAGTTAGCAAGT	
Dicer2 CHK_R	CCATCTTCCGGTCTGATTCTTC	
Vir-1 CHK_F	GCCAAAGTCCGGTATTCTTC	

Vir-1 CHK_R	TTCACGAGATCGTCAAGGTAA	
Dome CHK_F	AAACGGTGGCAAAATGAACT	
Dome CHK_ R	CATACAGCCGGCTTTCTTCT	
LacZ RNAi_F	TAATACGACTCACTATAGGGGTCGCCAGCGGCACCGCGCCTTTC	For double-stranded RNA construction and synthesis
LacZ RNAi_R	TAATACGACTCACTATAGGGCCGGTAGCCAGCGCGGATCATCGG	
AaEcR RNAi_F	TAATACGACTCACTATAGGGAAGCGAGGTTATGATGTTGCGAATG	
AaEcR RNAi_R	TAATACGACTCACTATAGGGTGAGGACGAGGACTGGGTGCC	
AaCaspar RNAi_F	TAATACGACTCACTATAGGGACCGCTATTGAAGATGTCGC	
AaCaspar RNAi_R	TAATACGACTCACTATAGGGCGGCGTTACGTCCAAATAGT	
