# Supplementary figures and images for: Dual functions of the Aedes aegypti ecdysone receptor in dengue virus replication and reproduction control
Source: Parasit Vectors. 2026 Mar 22;19:190. doi: 10.1186/s13071-026-07298-0 (PMC13130442; doi:10.1186/s13071-026-07298-0)

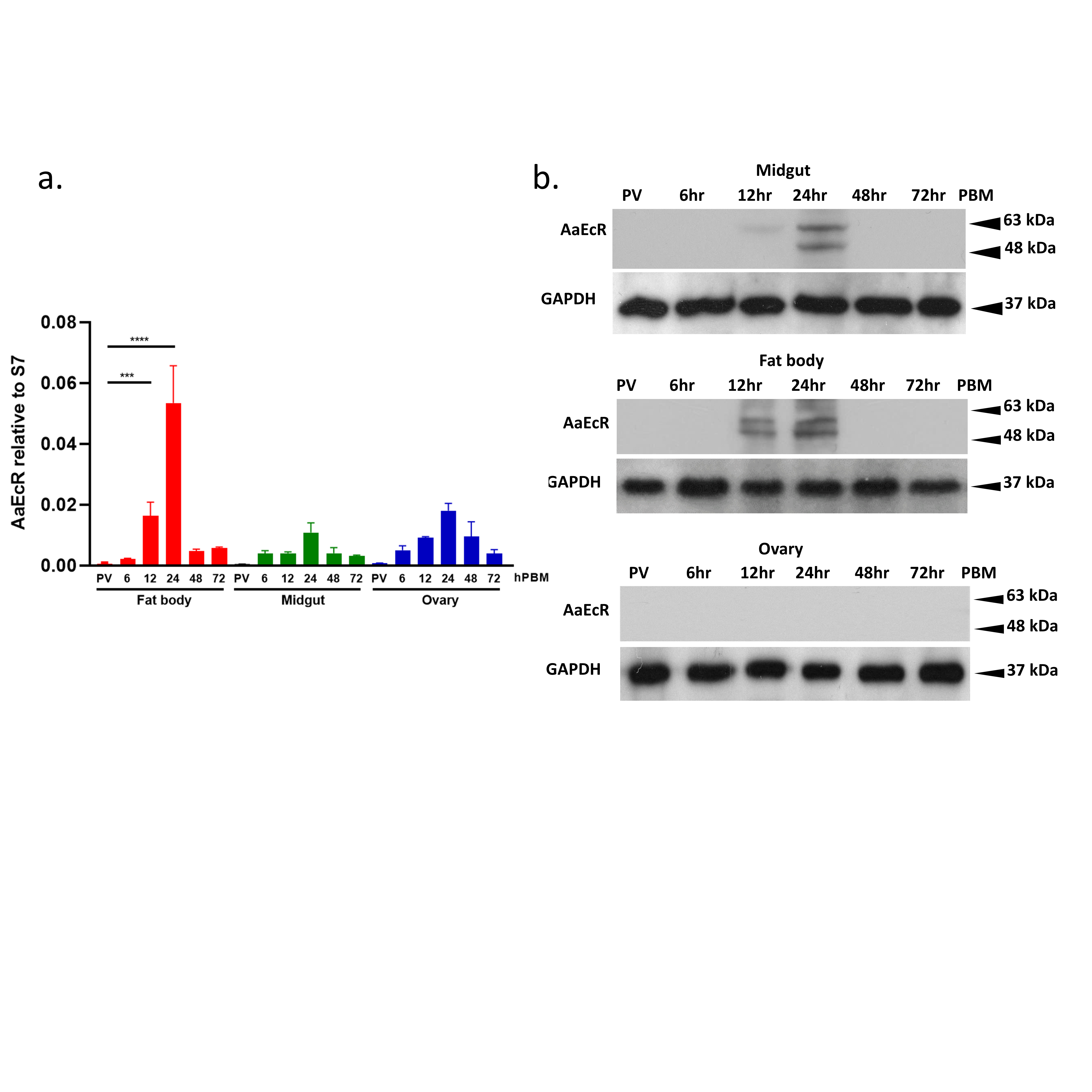

Supplement: Supplementary file 2 — Additional file 2. Figure 1. Temporal expression profile of AaEcR mRNA and protein in selected tissues of wild-type Aedes aegypti following a blood meal. Wild-type female mosquitoes (3–5 days old) were starved for 24 h prior to receiving a blood meal. Tissues including the fat body, midgut, and ovary were dissected at defined timepoints: pre-vitellogenic stage (PV), and at 6, 12, 24, 48, and 72 h post-blood meal (hPBM). a Total RNA was extracted from these tissues and reverse-transcribed into cDNA. Quantitative PCR (qPCR) was performed to assess AaEcR mRNA expression levels. Ribosomal protein S7 was used as the internal control for normalization. The data were analyzed by the Mann–Whitney U test (***P<0.001, ****P<0.0001). b Protein lysates from the fat body, midgut, and ovary were collected at the same timepoints and subjected to immunoblotting using a monoclonal α-AaEcR antibody [file 13071_2026_7298_MOESM2_ESM.tif]
